# 1454. Descriptive Epidemiology of *Candida auris* Infections in African-American population in Tertiary Care Center in Greater Detroit Area

**DOI:** 10.1093/ofid/ofad500.1291

**Published:** 2023-11-27

**Authors:** Erin Pollock, Avnish Sandhu, Vishakh C Keri, Catherine Maples, Hossein Salimnia, Teena Chopra

**Affiliations:** Wayne State University School of Medicine, Detroit, Michigan; Wayne State University , Detroit, Michigan; Wayne State University, Detroit, Michigan; Wayne State School of Medicine, Detroit, Michigan; Wayne State University, Detroit, Michigan; Detroit Medical Center, Wayne State University, Detroit, MI

## Abstract

**Background:**

The first reported *Candida auris* (*C. auris*) cases in United States, were associated with global travel. It has emerged as a nosocomial pathogen among critically ill patients with prolong hospital stay, patients in long-term-acute hospitals, and skilled nursing facilities. Given the increase in cases (59% in 2019 to 95% in 2021) and *C. auris* being multidrug-resistant, Centers for Disease Control and Prevention has declared it to be a pathogen of urgent public health threat. The purpose of this study is to describe the epidemiology of *C. auris* among patients in a tertiary care center in greater Detroit area.

**Methods:**

A retrospective cohort study was conducted among patients who tested positive for *C. auris* between January 2021 to January 2023. See Figure 1 flow chart of screening and culture method used to identify *C. auris*. Patients were defined as infected or colonized based on review of medical documentation. Data on demographics, length of stay, source of admission, treatment and susceptibility report were collected.

**Figure:**
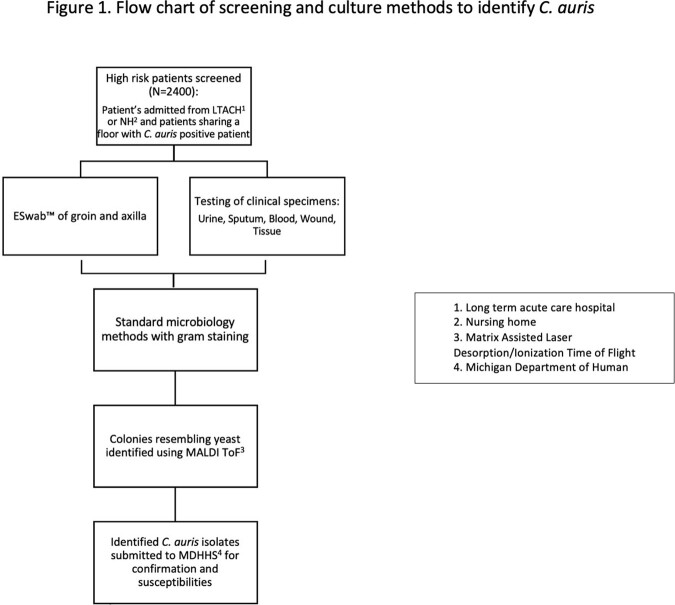

**Results:**

One hundred and eight patients tested positive for *C. Auris* with 10 infections and 98 colonizations. 90% of infected patients were in intensive care unit, 80% required a ventilator, 50% had fungemia and 40% died within 30 days of *C. auris* infection (Table1). Among the 4 patient that died, median age was 67.5, one patient had persistent *C. auris* fungemia due to endocarditis, one patient had decompensated cirrhosis with concomitant bacteremia and urinary tract infection with *C. auris*, one patient had *C. auris* mediastinitis with severe cardiac comorbidities, and one patient had abdominal aortic aneurysm repair developed ventilator associated pneumonia with *C. auris* and *Aspergillus Niger* complicated by severe acute respiratory distress syndrome. 80% of patients were treated with micafungin and one patient was treated with micafungin and amphotericin B given *C. auris* endocarditis (Table 1). Voriconazole was used for treatment in one patient due to concomitant *Aspergillus* infection (Table 1). See Table 2 for minimum inhibitory concentration for 8 of 10 isolates.

**Figure:**
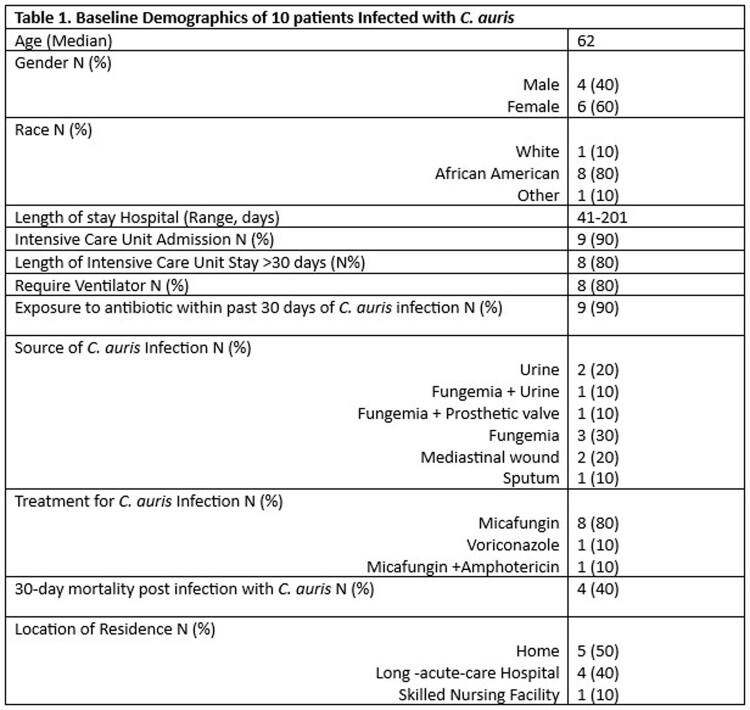

**Figure:**
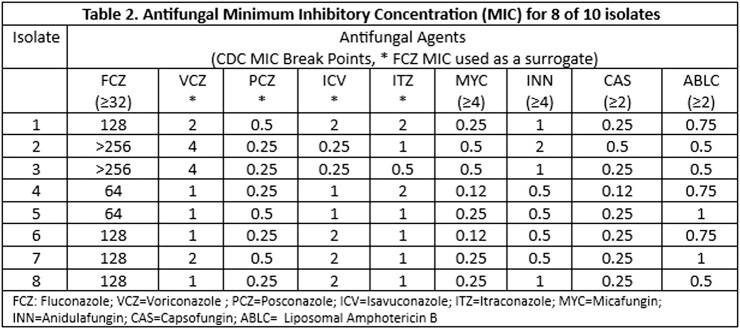

**Conclusion:**

*C. auris* is an emerging pathogen that can colonize the skin and has a potential of causing invasive infections leading to high morbidity and mortality.

**Disclosures:**

**All Authors**: No reported disclosures

